# Pharmacological manipulation of transcription factor protein-protein interactions: opportunities and obstacles

**DOI:** 10.1186/s13619-015-0015-x

**Published:** 2015-03-12

**Authors:** Frank Fontaine, Jeroen Overman, Mathias François

**Affiliations:** Division of Genomics of Development and Diseases, Institute for Molecular Bioscience, The University of Queensland, 306 Carmody Road, St Lucia, QLD 4072 Australia

**Keywords:** Transcription, Screening, Proteomics, Interactome, Pharmacology, Specificity, Cancer, Genomics

## Abstract

Much research on transcription factor biology and their genetic pathways has been undertaken over the last 30 years, especially in the field of developmental biology and cancer. Yet, very little is known about the molecular modalities of highly dynamic interactions between transcription factors, genomic DNA, and protein partners. Methodological breakthroughs such as RNA-seq (RNA-sequencing), ChIP-seq (chromatin immunoprecipitation sequencing), RIME (rapid immunoprecipitation mass spectrometry of endogenous proteins), and single-molecule imaging will dramatically accelerate the discovery rate of their molecular mode of action in the next few years.

From a pharmacological viewpoint, conventional methods used to target transcription factor activity with molecules mimicking endogenous ligands fail to achieve high specificity and are limited by a lack of identification of new molecular targets. Protein-protein interactions are likely to represent one of the next major classes of therapeutic targets. Transcription factors, known to act mostly via protein-protein interaction, may well be at the forefront of this type of drug development. One hurdle in this field remains the difficulty to collate structural data into meaningful information for rational drug design. Another hurdle is the lack of chemical libraries meeting the structural requirements of protein-protein interaction disruption.

As more attempts at modulating transcription factor activity are undertaken, valuable knowledge will be accumulated on the modality of action required to modulate transcription and how these findings can be applied to developing transcription factor drugs. Key discoveries will spawn into new therapeutic approaches not only as anticancer targets but also for other indications, such as those with an inflammatory component including neurodegenerative disorders, diabetes, and chronic liver and kidney diseases.

## Introduction

The concept of pharmacological manipulation of protein-protein interaction (PPI) was clearly demonstrated with taxane anticancer drugs, paclitaxel and docetaxel, identified half a century ago. These compounds of natural and semisynthetic origins block microtubule depolymerization and mitosis in tumor cells via a mechanism of stabilization of tubulin heterodimers, eventually leading to apoptosis [1]. In 2014, the market for taxane anticancer drugs was valued at around US$6 billion for United States, Japan, and Europe [2]. It is now widely admitted that a large majority of the estimated 3,000 druggable proteins [3] function as complexes within a network of interactions [4-6], rather than acting as single effectors. As a result, the modulation of protein-protein interactions by small organic molecules, so-called “protein-protein interaction disruptors” or PPIDs, offers innovative therapeutic avenues [7,8].

**Figure 1:**
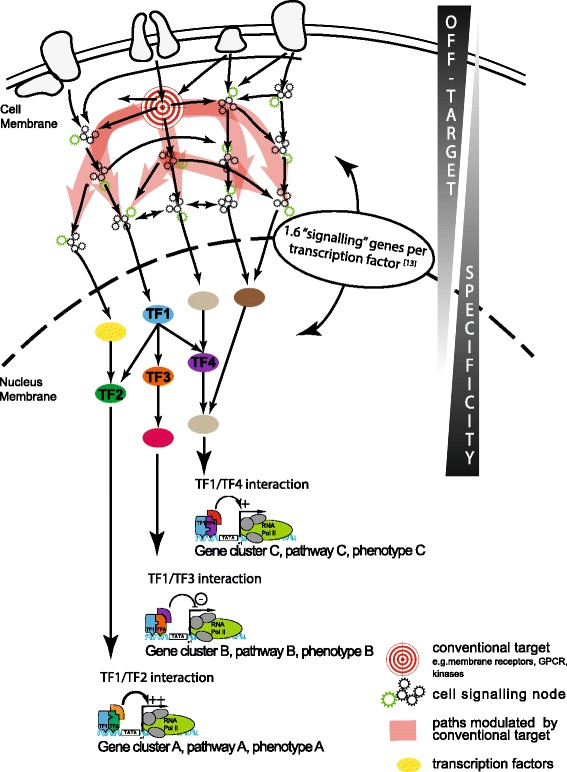
**Advantages of targeting transcription factor protein-protein interactions over conventional targets.** Targeting upstream cell-signaling nodes (e.g., kinase) lacks specificity because of the interconnected nature of cell signaling and its numerous feedback loops. Targeting transcription factors, downstream effectors of cell signaling, able to control specific gene groups via specific protein associations, is likely to be a more specific approach [13].

Within the field of PPIDs’ discovery, particular types of protein-protein interactions are easier to target than others, such as transmembrane, cytoskeleton, and mitotic proteins, as well as nuclear receptors, with exciting anticancer and anti-infective indications. Nuclear proteins such as transcription factors (TFs) still remain a challenge to manipulate using chemical-based strategies. Pharmacological management of transcription factors is usually achieved in more classical ways, including inhibition of upstream phosphokinase (lack of specificity) [9,10] or via mimicking endogenous ligands (nuclear receptors) [11,12]. Despite major hurdles in specifically targeting transcription factor activity, their central role in controlling cell signaling and their mode of action as dynamic complexes position them at the forefront as targets of choice for PPIDs (Figure 1).

In this review, we aim to reposition the study of transcription factor biology in its historical context and from there to weigh the impact of recent methodological and conceptual breakthroughs on future developments. We will briefly discuss strategies to develop pharmacological manipulation of transcription factors, focusing on protein-protein interactions and small compounds.

## Transcription factors: background and recent developments

### Basic principles of eukaryotic transcription

Eukaryotic transcription is a complex mechanism classically requiring core promoter factors and gene-specific transcription factors, which assemble cooperatively on cognate DNA elements located on the promoter, upstream of the gene coding region, marked by the ATG “start” codon [14]. Core promoter factors and RNA polymerase II assemble on the gene promoter at the transcription start site, consisting in a broad CpG island or a TATA box [14,15]. Gene-specific transcription factors, conversely to core promoter factors, display specificity towards sets of target genes [16,17]. They bind to DNA elements that may be within the proximal promoter (<5 kb) or in the remote part of the chromatin thousands of nucleotides away. Co-regulator proteins in turn help connect DNA-binding TFs to the general transcriptional machinery or locally modify chromatin structures to facilitate transcriptional activation [18,19]. RNA polymerase II is then recruited via the mediator, a large protein complex required for almost all transcriptions of RNA polymerase II promoters, finally initiating gene transcription [20].

Two other groups of transcription factors, not directly involved in the final steps of transcription initiation, yet pivotal in transcriptional regulation, should be mentioned. These are pioneer transcription factors that can bind directly to condensed DNA and “chromatin-associated” high-mobility group (HMG) proteins, very close to pioneer transcription factors, which bind to DNA with low sequence specificity, except for the Sox sub-family [21,22]. Pioneer transcription factors (for example, CTCF, GATA) and HMG proteins (for example, HMGA, HMGB) modulate chromatin architecture and are more abundant in cell nuclei compared with “gene-specific” transcription factors [21,23-25]. Pioneer transcription factors and HMGA and HMGB proteins are able to open up histone-occluded chromatin, higher-order chromatin structures, or repressor complexes [21,23]. The Sox sub-family of HMG proteins, on the other hand, is not considered able to do so, according to a new functional classification based on transcription factors’ effect on chromatin architecture [26,27]. In this new classification, based on computational analysis of DNase I-digested genome sequence, the Sox sub-family of HMG proteins is positioned in an intermediate group between pioneer and gene-specific transcription factors, the “migrant” transcription factors, unable to open condensed chromatin [26,27]. Both pioneer transcription factors and HMG proteins—including Sox—control the timing of transcription during development and cell differentiation and are therefore key factors in global transcriptional regulation [21,23,28].

Direct chemical modulation of TFs’ activity is an expanding field, as already demonstrated with nuclear receptors, exploiting their ability to capture small endogenous ligands, in structurally well-defined binding sites [29]. In hormone-dependent cancers, like breast and prostate cancers, drug resistance to nuclear receptor antagonists is a frequent problem that may be avoided by targeting accompanying pioneer factors [30,31]. Not only for this reason but also because of their key role in early development and cancer, pioneer transcription factors as well as HMG proteins are the next two groups likely to open new therapeutic windows [21,32].

### TF functions: current state of knowledge

The first transcription factor ever discovered was the simian viral repressor SV40 T antigen (simian virus rumor antigen) in the late 1970s [33]. In the early 1980s, the first human transcription factor was discovered, the potent activator zinc finger transcription factor Sp1 [34]. Twenty years later, by the time the human genome sequence was published, JC Venter and collaborators predicted the total number of transcription factors to be 1,500, making it the second most common molecular function for a gene protein product after enzymes [35]. However, of this predicted 1,500 protein-strong transcription factor proteome, less than 5% were purified and characterized by 2001 [36]. Projects of automated annotation of genomic functional elements, like the ENCODE consortium initiated in 2003 (histone marks, transcription factor binding, chromatin regulators, RNA-binding proteins, etc.), FANTOM5 (regulatory elements such as enhancers), or, in 2010, the smaller Dragon database (transcription co-regulators and transcription factor-interacting proteins, TcoF-DB), have started to identify, locate, and sequence functional elements [37-39]. These ventures have not only revolutionized our understanding of genome structure and function but have also given us the false notion that we have gained knowledge in the biochemical properties of TFs. Since 2001, the predicted number of human TFs has been refined to about 1,700–1,900, and less than 200 co-regulators. However, only a meager 62 TFs have been functionally validated, not just annotated according to their DNA-binding coordinates [13,38]. Expanded experimental opportunities to perform quantitative study of TF biochemical properties, as measured with either cell-free (requiring recombinantly expressed/purified TF) or reporter-based technologies, are only available for a restricted number of TFs [13,40]. Without this level of experimental “tractability”, further studies required for target validation are impossible.

The discipline of developmental biology has contributed the most to the identification and characterization of TFs, prevalently in non-human organisms, with observations not necessarily applying to human orthologues [13]. Consequently, it is not surprising that of the 12% of transcription factors (consisting of a DNA-binding domain and a transactivation domain) directly responsible for diseases or syndromes, the largest portion involves developmental defects [13,41-43]. The second and third fields of investigation that have contributed the most to transcription factor discovery are the study of cell signaling and cell metabolism [13]: cell signaling because the study of interconnected signaling pathways always converges at transcription factors [17] and cell metabolism because metabolic processes are instructed by nuclear receptors, capable of directly activating gene transcription upon binding of endogenous hormone ligands [44]. In summary, the sheer number of studies published on human transcription factors, over 90,000 in 30 years (for the top 20 transcription factors), and the rapid development, in the last decade, of electronic annotations generated by sequence search algorithms, together conceal the fact that we have very limited knowledge on how human TFs function [40].

### TF proteome: recent landmark advances in profiling methods

DNA microarray and qPCR analyses have confirmed that transcription factors are consistently expressed at lower levels than other genes across 32 human tissues [45]. This seems logical as a single transcription factor can trigger the generation of many copies of mRNA from a single target gene. In the nuclear compartment, however, there is seldom but direct evidence that the local concentration of TFs can vary at least several fold. HMG proteins, HMGA2 and HMGB2 for instance, are three to six times more abundant than other TFs in human epithelial cell line nuclear extract [24]. Sox2, another HMG protein, reaches almost millimolar levels in the nucleus of embryonic stem cells [25]. From a law of mass action perspective, keeping gene-specific transcription factor abundance low, i.e., spanning the Kd values of the best binding sites, could prevent them from binding to lower affinity sites, with an undesired transcriptional effect [46]. Similar observations were made in bacteria [47]. Conversely, for TFs at the top of the hierarchy, like pioneer TFs that control multiple distant genomic areas, a higher nuclear concentration is required for rapid 3D diffusion [25,47]. At the whole-tissue level, the low abundance of TFs, along with nucleus compartmenting, and the difficulty to separate DNA-binding proteins from genomic DNA explain why transcription factor affinity purification and pull-downs have been technically challenging [36,48,49]. In 2007, however, with the generalized usage of the genome-wide protein binding assay “ChIP-seq” (chromatin immunoprecipitation sequencing) later on combined with protein mass spectrometry (“rapid immunoprecipitation mass spectrometry of endogenous proteins”, RIME), functional study of transcription factors dramatically accelerated [50]. Further, a new remarkable approach has been developed that involves transcription factor affinity purification with a DNA concatemer composed of multiple tandem repeats of a specific responsive element. The method developed over 20 years ago was not initially able to improve on standard affinity chromatography methods [36,51]. In a recent development, a DNA concatemer made of the tandem juxtaposition of 100 selected transcription factor-responsive elements allowed authors to identify, and purify, almost 900 transcription factors from the nuclear extract of 11 different mammalian cell lines, as well as measure their DNA binding activity in one single purification step [24]. This new methodology in association with new-generation rapid MS-based protein identification brings transcription factor proteomics to the throughput level of RNA-seq (RNA-sequencing) [24]. DNA concatemer pull-down analysis is now able to measure proteome-wide changes in transcription factor binding activity in response to drug treatment in any cell line or tissue. These recent methodological breakthroughs, along with single-molecule imaging of hundreds of millisecond span-lived nucleus enhanceosomes (transcription factor assembly on their cognate DNA target sites), will shed light onto molecular mechanisms of transcriptional regulation [24,25]. As a consequence, refined classification of transcription factors based on nuclear stoichiometric abundance, association-dissociation kinetics, co-regulating partners, and the type of DNA they are bound to (methylated, condensed) will emerge and will challenge the existing classification [52,53]. These recent breakthroughs undoubtedly lift major impediments for key players in drug development, in the study of transcription factors as potential molecular targets.

## Transcription factors as molecular targets

### Basic concepts of target protein “druggability”

Over the last two decades, drug discovery research has been transitioning from searching for compounds active against diseases but with unknown targets to screening for specific inhibitors of disease-relevant proteins [54]. The basic concept of target druggability was coined in 2002 by Hopkins and Groom and mostly remains an empirical issue, constantly reassessed with each new attempt to find drugs [3].

All drugs currently available on the market target less than 500 proteins, of both pathogen and human host origins. More than 50% of these drugs target three types of proteins only, class I G protein-coupled receptors (GPCR; 27%), nuclear receptors (13%), and ion channels (13%) [55,56]. Advantageous physicochemical properties for drug binding can be estimated for any disease-relevant protein: simply put, the presence of a deep hydrophobic pocket that is large enough to allow for high-affinity binding of a drug-like molecule, in turn, able to modulate the target’s “activity” [57]. To some extent, off-target adverse effects can also be predicted based on the same binding site characteristics, as well as knowledge accumulated from decades of clinical trials and emerging inter-disciplinary systems biology [58]. Other, less tangible aspects of druggability are much harder to predict, including any “experimental hurdles” and unforeseen adverse effects, until trialed [57].

### TF “druggability” and PPIs

#### General problem of targeting protein-protein interactions

**Figure 2:**
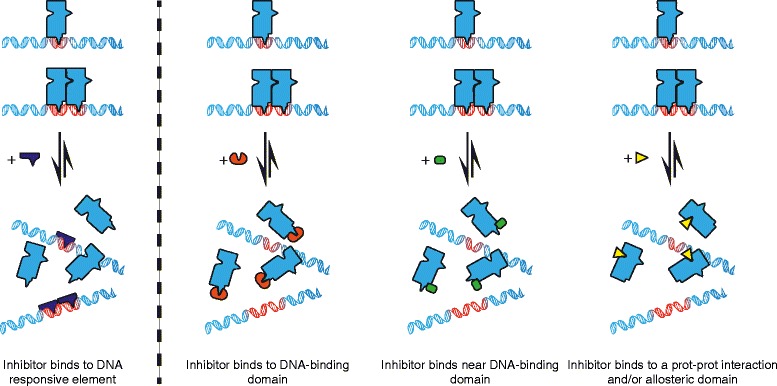
**Mode of action of transcriptional activation inhibitors.** Small compounds directly inhibiting transcriptional activation can act by targeting DNA (blue), or transcription factors/co-activators, either preventing DNA binding (red and green) or altering protein conformation or protein-protein interaction (yellow). Inspired from [16].

Application of basic concepts of target “druggability” to TFs highlights a number of important challenges. Modulation of TF activity can be achieved via a few different approaches, including direct or indirect modulation of their own expression, modulation of their DNA binding activity, and modulation of their ability to interact with partner proteins (Table 1). We will focus this discussion on protein-protein interface inhibition by small compound antagonists. Boundaries between small compounds, peptides, and peptide mimetics are blurred; small compound PPI disruptors (PPIDs) are heavier, more hydrophobic, more rigid, and more planar than conventional small compound drugs, and peptide motifs are often included in their structure as part of their rational design [59,60]. Most transcription factors form homo- or heterodimers to be part of a larger complex subunit that operates in a cooperative fashion (Figure 2). Whether DNA binding affinity and specificity for cognate DNA gain from TF PPI [61,62] or not [63] remains a controversial question. Even so, disturbing the dimerization/partner recruitment of a crucial TF to exert influence on gene expression is already a proven effective strategy for nuclear receptors with a protein interface centered around a well-defined binding pocket [11,12,16]. For other transcription factors, targeting protein-protein interactions with 10-Å-long small molecules is a challenging task owing to the large, diffuse, and polished surface areas involved in protein-protein binding (1,500–3,000 Å^2^ compared to a few hundreds to a thousand for a “classic” binding pocket) and the lack of obvious concave binding pockets at many protein-protein interfaces or allosteric sites [64]. In addition, high-throughput screening technologies to identify compounds able to disrupt protein-protein interfaces are not routinely available for various reasons [65]. The main limitation of *in vitro* homogenous protein-protein interaction assays is access to sufficient amounts of the functional proteins themselves. Even so, post-translational modifications fundamental for protein functionality can be lacking in recombinant proteins. Finally, purification of TFs is notoriously difficult, as they tend to bind to genomic DNA. Despite these technological limitations, a eukaryotic cell-free protein expression system coupled to AlphaScreen-based measurement of protein-protein interaction has been described, enabling rapid mapping of protein interaction networks and high-throughput screening for protein-protein interaction inhibitors [66]. This study has opened the way to target TFs as part of a network of interactions rather than addressing individual PPIs specific to a few particular TFs.

**Table 1:** **Summary of direct TF inhibition strategies**

**Mode of action**	**Class of drug**	**General benefits**	**General drawbacks**	**Examples/indication**
Inhibition of TF gene expression	Antisense mRNA, siRNA	Selectivity	Antisense mRNA and duplex unstable, poor cellular uptake, pro-inflammatory, resistance mechanisms (gene over-expression)	K-Ras/cancer [67,68]
Binding to TF DNA-binding domain	Decoy oligonucleotide	Selectivity	Low bioavailability, short half-life, poor cellular uptake	Sp1, AP-1, STAT3, Ets-1/cancer [69]
	Small molecule	Bioavailability	Off-target effects	Human androgen receptor/cancer [70]
	Small peptides and peptidomimetics	Less or no side effect	Low bioavailability, unstable, pro-inflammatory	STAT3/cancer [71]
Disruption of protein-protein interaction	Small molecule	Bioavailability	Off-target effects	cMyc/Max, Max/Max, HDM2/p53, Bcl/Bax/cancer [17,60,72,73]
	Small peptides and peptidomimetics	Less or no side effect	Low bioavailability, unstable, pro-inflammatory	STAT3, MDMX/p53/cancer [74-76]

Although protein-protein interfaces are large, studies show that amino acids usually found at the center of the interface and representing less than half of its surface contribute to most of the binding energy [77]. In the past 5 years, there has been remarkable progress in identifying small molecules that bind to these interfaces. Empirical experience shows however that compounds binding to the aforementioned “core” amino acids alone are not high-affinity inhibitors and need additional sources of affinity, like pockets not naturally engaged by protein partner(s) [64].

A protein involved in protein-protein interactions often uses the same interface to bind “promiscuously” to several protein partners [78]. In turn, one can easily predict that a small molecule designed to interact with such interface will also show some promiscuity for partner proteins interacting with the target protein initially selected for its design. Pharmacological promiscuity is considered to be a bad omen in classical drug discovery projects, focused for example on enzymes or G protein-coupled receptors (the “one drug-one target” paradigm), leading to adverse drug reactions and obscuring pharmacodynamics effects in animal models. If protein-protein interactions are to be “drugged” in the near future, the aforementioned “promiscuity” concept will have to be redefined in a more restrictive manner for small molecules that disrupt these interfaces. Here, the concept of selectivity requires a paradigm shift. For a PPID to be functional, the small-molecule inhibitor will have to “excise” its target protein “out” of an interaction network, meaning that some promiscuity towards surrounding partner proteins may in fact be beneficial. This will prevent redundancy mechanisms that are often in place to make up for the loss of activity of a specific transcription factor.

#### Risks associated with targeting TFs, the “focal point” of cell signaling pathways

“Drugging” transcription factors, the “point of convergence” of multiple signaling pathways, in turn controlling multiple target genes, is largely considered a perilous task, due to the broad consequences of modulating their activity. Human genome analysis has recently revealed that cell signaling networks consist of approximately 3,000 genes, 1,800 involved in intra-cellular signaling (kinases and phosphatases implicated in protein phosphorylation) and 1,300 in cell-cell communication [79]. In comparison to the 1,700 to 1,900 human transcription factor proteome, this amounts to an average “ratio” of approximately 3,000/1,900 ≈ 1.6 “signaling” genes per transcription factor. This ratio, not as “high” as suspected, is likely to leave room to redundancy mechanisms for most TFs [13]. Similar concerns were raised 20 years ago about protein kinases “serving critical cellular functions” and “difficult to target specifically”, when the first-generation tyrosine kinase inhibitor “Gleevec” was discovered and fast-tracked to market 10 years later [80]. Today, 10% of experimental and marketed drugs are targeting serine/threonine and tyrosine protein kinases, generating a US$20.2 billion market in 2014 [3].

Very recently, in a lymphoblastoid cell line, the expression of 59 transcription factors and chromatin modifiers was independently knocked down (by at least 50%, using small interfering RNAs) and down-regulated genes were identified in three independent microarray experiments [81]. The number of genes differentially expressed approximately ranged from 40 to 4,000, depending on the knock down experiment. Microarray data were compared to data obtained with negative control siRNA, and the reduced list was then cross-checked with ChIP-seq and DNase-seq binding maps of the aforementioned transcription factors and chromatin modifiers [82]. Binding of a knocked-down transcription factor was deemed functional only if a binding site was within 10 kb of the transcription start site of an affected gene [81]. Surprisingly, only 11% of differentially expressed genes could be associated with any decrease of TF binding, and the median level of down-regulation for these target genes was less than 10% compared to negative control. This work illustrates our current lack of understanding of TF redundancy mechanisms and defines the need to develop proper “biochemical” functional assays *in vitro* and *in vivo*.

### Modes of intervention

#### Targeted indications

##### Cancer

**Figure 3:**
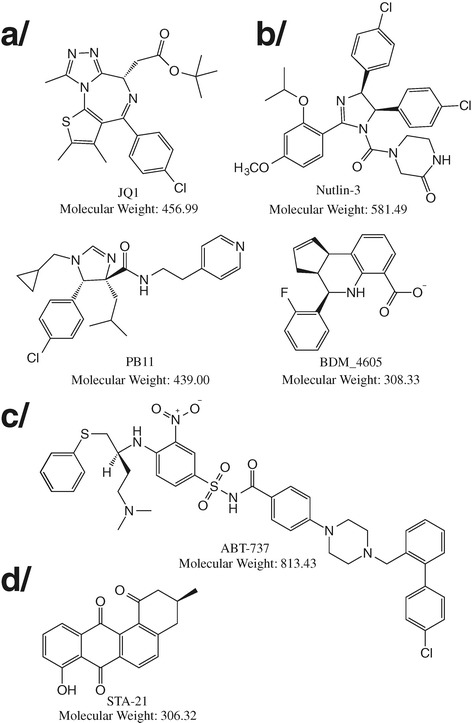
**Transcription factor protein-protein interaction disruptors: heavier, more hydrophobic, and more rigid than regular drugs.** Anticancer indications: **a/** BRD4/histone interaction disruptor: BRD4 binds to acetylated lysine residues in histone tails, which act as activation markers for gene expression. (+)-JQ1 was modeled to fit a hydrophobic cavity in the BRD4 domain that accommodates acetylated lysines. Potential indications are squamous cancer and acute myeloid leukemia [86]. **b/** HDM2(MDM2)/p53 interaction disruptors: Human or mouse double minute 2 protein binds to tumor suppressor p53, increasing its degradation. Tetra-substituted imidazole, Nutlin-3, disrupts complexes with nanomolar affinity [87]. Subsequent attempts at computational optimization based on existing PPI descriptors and X-ray crystallography have generated compounds with low/sub-micromolar affinities PB11 and BDM_4605 [60,72]. Potential indications are all p53-related cancers. **c/** Bcl-2(Bcl-xL)/Bax(Bak) interaction disruptors: Bcl-2 or Bcl-xL binds to the BH3 (Bcl2-homology 3) domain of pro-apoptotic Bax and Bak proteins, preventing apoptosis. BH3-mimetic ABT-737 disrupts complexes with sub-micromolar affinity, inducing apoptosis. Potential indications are small-cell lung carcinoma and lymphoma [73]. Other indications: **d/** STAT3 is a latent cytoplasmic transcription factor transducing signals from the cell membrane to the nucleus. STAT3 can dimerize via reciprocal interaction of its Src homology 2 domain (SH2), upon phosphorylation of a conserved tyrosine within the SH2 domain. STA-21 inhibits cytokine-dependent nuclear translocation of Stat3 in normal human keratinocytes *in vitro* by impeding STAT3 DNA binding and dimerization with mid-micromolar potency [88].

It is widely accepted that most anticancer chemotherapies are marred with taxing side effects and risks of relapse with resistant tumors. Archaic DNA-alkylating cisplatin, for instance, the first member of platinum-containing anticancer drugs approved by FDA more than 35 years ago, displays acute and indiscriminate cytotoxicity, not to mention common relapse with cisplatin-resistant tumors. Yet, it is still, today, a cornerstone of modern anticancer treatment. Better, more discriminating treatments are urgently needed [83]. With the discovery of the first oncogenes, starting with the chicken retrovirus gene *sarc*, more than 40 years ago, it was rapidly identified that the normal counterparts of oncogenes would be transcription factors whose proper function was the control of physiological cell growth. Their modulation would in turn profoundly affect the course of growth-related diseases such as cancer [84]. The number of transcription factors listed as targets of choice for cancer therapy, able to modulate tumor growth and/or metastasis, has steadily increased in the last decade (Figure 3). Most promising research projects are targeting amongst others BRCA1, a tumor suppressor protein involved in DNA repair; MYC/MAX heterodimerization, involved in cell proliferation and differentiation suppression; *FOXM1*, a transcriptional repressor involved in chromosomal segregation and genomic stability (most intensively investigated with tumor suppressor p53); as well as FOXA1, a transcription factor controlling the expression of other genes involved mostly in hormone-dependent breast cancer [16,43,85].

##### Other indications

Pharmacological manipulation of transcription factor protein-protein interaction is still in its infancy. Transcription factors are first and foremost seen as oncogene counterparts, controlling cancer-related cell growth disorders. A handful of potential non-cancer indications have been proposed, however, with promising results, for example with STAT protein modulators. STAT proteins are a family of transcription factors shuttling between the cytosol and the nucleus, depending on their JAK kinase-dependent phosphorylation status, linked to cytokine membrane receptors. In 2005, inhibition of STAT3 dimerization was already predicted as an alternative choice to targeting upstream phosphokinases [10]. This has now become a reality, with psoriasis identified as a potential indication for modulators of STAT3 dimerization (Figure 3) [88]. A few other indications have also been identified for protein-protein interaction modulators, like anti-angiogenesis for p300-HIF-1α [89] as well as type II diabetes for CREB (cAMP response element-binding protein)-CBP (CREB-binding protein) interaction [90].

### Marketed drugs with documented off-target effects towards TFs

To our knowledge, there is no compiled list of marketed drugs with documented off-target effects towards transcription factors, except for nonsteroidal anti-inflammatory drugs (NSAIDs) [91]. It has been known for over a decade that the antineoplastic properties of some NSAIDs are due to both COX-related and COX-independent modes of action. COX-independent antineoplastic modes of action include modulation of two transcription factors: activation by direct binding of antineoplastic nuclear receptor PPARγ and inhibition of transcription factor NFκB-dependent gene expression [92,93]. A body of evidence is developing that identifies a possible link between the two modes of actions via “receptor-interacting-protein-of-140-kDa” (RIP140), a co-repressor of PPARγ as well as a co-activator of NFκB-mediated inflammatory gene expression. Targeting RIP140-protein interactions occurring along these pathways may open new therapeutic avenues for not only indications with an inflammatory component, such as cancer, but also Alzheimer’s disease, type II diabetes, and chronic liver or kidney diseases [94-96].

The depletion of physiologically important prostaglandins due to cyclooxygenase inhibition precludes the long-term use of antineoplastic NSAIDs for cancer chemoprevention. Conversely, no side effect due to alteration of PPARγ and NFκB-dependent gene expression has been documented thus far, arguing for the safety of pharmacological manipulation of TFs, either via classic approaches or via PPIDs.

## Methodological obstacles to screening and design of TF modulators

### TF recombinant expression and purification

Affinity tag-based protein purification methods differ largely in terms of purity, yield, capacity, and cost, and transcription factors are no exception to this rule [97]. However, eukaryotic transcription factors are also renowned for being difficult to express and purify. Because of the importance of post-translational modifications [98], TFs should always be expressed in eukaryotic systems, e.g., CHO, HEK mammalian cells, or insect cells. If yield is poor in native conditions (low to sub-milligrams per liter), it should be compared to yield in denaturing conditions, using lysis, washing, and elution buffers supplemented with 8 M urea. A comparatively poor yield in native conditions is often attributed to sterical obstruction of the affinity tag in the protein native conformation. According to the authors’ own experience, however, it is rarely the case for TFs, which seem to have a rather ill-defined native conformation, even for very small tags. Instead, solubility issues and co-precipitation with genomic DNA are the two most common problems encountered. Single-step glutathione-S-transferase (GST) purification from a baculovirus/insect cell expression system seems to correct both issues, providing the best ratio of native to denaturing purification yields and the best cost compromise. Several fusion proteins have been shown to increase protein solubility, including 26-kDa GST, the maltose-binding protein, and the Z-domain from protein A [99-101]. Two-step affinity purifications, on the other hand, are often marred with problems of elution buffer incompatibility, e.g., the immobilized metal ion affinity chromatography (IMAC) elution buffer is not optimal for GST binding or vice versa. Likewise, addition of a secretion signal like honeybee “melittin” has been attempted in several occasions but did not yield any particular improvement to the method [102].

Finally, for purified transcription factors with severe stability issues, storage at 4°C on GSH beads may preserve them from aggregating for a few weeks. With a cleavage site included either for the human rhinovirus 3C protease or the cysteine protease of the tobacco etch virus [103], the GST tag can be easily removed for functional assays (pull-down, affinity purification-mass spectrometry) or crystallization.

### Access to suitable annotated small compounds libraries

Screening libraries of synthetic molecules has been productive against traditional drug targets, such as ligand-gated ion channels, kinases, and G protein-coupled receptors [104,105]. More success may be recorded in the forthcoming years for enzymes and receptor ligands identified using metabolomics profiling, i.e., profiling of small molecules occurring naturally in an organism [3]. Conversely, for antimicrobial targets and targets identified from genomic studies (including DNA recombination, sequencing, and bioinformatics studies), screening productivity has been problematic and is expected to remain so if some paradigms are not challenged [3]. Because total chemical space is estimated to be greater than 10^60^ molecules for a molecular weight below 430 g/Mol^−1^ (or Dalton), chemical libraries have to be dramatically biased towards biological targets to reduce size and improve odds of random hits. This bias is mainly obtained by mimicking “biogenic” natural products [106]. Combinatorial chemistry has been an essential part of drug discovery for the last 30 years, based on the assumption that increasing the size and diversity of libraries by systematic combination of basic chemical motifs bound by Lipinski’s rule of five was the best approach. For more than a decade now, comparisons of combinatorial chemistry libraries with approved drugs and natural products have repeatedly pointed out a severe lack of chirality as well as structural rigidity of combichem libraries, the latter widely regarded as a prerequisite for tight binding of small molecules to protein-protein interfaces [107,108]. This explains at least in part why, today, only a small number of low-molecular-weight inhibitors of protein-protein interactions is available [109]. In the last 5 years, however, new rational drug design methods have emerged, allowing cost-effective assembly of chemical libraries biased towards protein-protein interaction inhibitors (Figure 3). New strategies employing machine learning based on known inhibitors, multicomponent reaction chemistry (also called “one-pot synthesis”) able to generate structural complexity in a single step, and associated with more classical fragment-based drug discovery approaches will soon generate readily accessible diversity libraries [59,60,72,110]. However, whether these libraries will generate higher hit rates for protein-protein interaction inhibitors remains to be seen.

### Access to high-throughput mapping of protein-protein interactions

Until recently, protein-protein interaction assays were amongst the most difficult biochemical assays to deploy in a molecular biology laboratory, requiring expensive equipment, expert skills, and time. Affinity purification-mass spectrometry (AP-MS) and yeast two-hybrid screening were the only two platforms available until the advent, 5 years ago, of cheaper technologies based on either fluorescence (fluorescence anisotropy, Förster resonance energy transfer, homogenous time-resolved fluorescence, AlphaScreen, and single-molecule fluorescence), proximity association of fragments into a functional reporter (a fluorophore or an enzyme), or label-free assays (surface plasmon resonance, isothermal titration calorimetry) [111-113]. The most common limitation is the possible interference of tags used in fluorescence-based or fragment proximity-based assays with protein folding or ability to interact with partner proteins.

In some instances, protein intra-cellular movements or translocations observed during signal transduction have been considered an acceptable surrogate to screen for inhibitors of protein-protein interaction, which is playing a key role in cell signaling [114,115]. This cannot be applied to intra-cellular protein-protein interactions of transcription factors, except if transcriptional activity is regulated by trafficking partner proteins to and from the cytoplasm, as is the case for SOX proteins for instance [116].

## Conclusion

The number of protein-protein interactions that comprise the human interactome is estimated at 650,000 PPIs [117]. Only a minute fraction of these are known and only a handful of low-molecular-weight disruptors (PPIDs) have been identified, displaying activities in the low-to-mid-micromolar concentration range. Protein-protein interactions are likely to represent one of the next major classes of therapeutic targets, with PPIDs showing great potential for further optimization, both in terms of potency and specificity.

New protein-protein interaction assays, ranging from fluorescent-based assays to genomic-wide RIME, as well as recent advances in TF proteomics profiling and dynamic intra-nuclear visualization methods, can now be deployed cost-effectively in non-specialist laboratories (developmental biology, regenerative medicine, microbiology, etc.). The timely convergence of all these affordable methodologies, amenable to fairly small sample size such as tissue biopsies, MACS sorted cells (magnetic activated cell sorting), and cultured cells, combined with advances in genome editing technology, will accelerate the identification of novel targets and the development of new compounds.

High-resolution NMR and X-ray crystallography of protein complexes can now be used as templates for the virtual screening of chemical databases, to identify the so-called “hot spot” binders. However, translation of structural knowledge (protein complexes, natural products, or peptidomimetic inspirational scaffolds) into rational drug design still remains a difficult task. Recently, new methodologies based on machine learning, *in silico* pharmacophore-based and *in silico* anchor-biased screenings, as well as stereoselective and one-pot chemical synthesis have led to rapidly increasing hit rates.

The era of small-molecule inhibitors of protein-protein interactions has only just began, and this is even more so the case for transcription factors.

## Abbreviations

PPID: Protein-protein interaction disruptor
TF: Transcription factor
HMG: High-mobility group
ChIP-seq: Chromatin immunoprecipitation sequencing
GPCR: G protein-coupled receptors
RIME: Rapid immunoprecipitation mass spectrometry of endogenous proteins
NSAID: Nonsteroidal anti-inflammatory drug
AP-MS: Affinity purification-mass spectrometry
RNA-seq: RNA-sequencing
